# Observational, prospective, phase 4 study in patients with first‐line recurrent and/or metastatic squamous cell carcinoma of the head and neck treated with cetuximab and platinum‐based therapy: DIRECT

**DOI:** 10.1002/cnr2.1467

**Published:** 2021-06-22

**Authors:** Joël Guigay, Emmanuel Chamorey, Gautier Lefebvre, Maciej Rotarski, Jean‐Philippe Wagner, Emmanuel Blot, Marc Alfonsi, Audrey Seronde, Jeltje Schulten, Frédéric Peyrade, Christophe Le Tourneau

**Affiliations:** ^1^ Comprehensive Cancer Center Antoine Lacassagne, FHU OncoAge Université Côte d'Azur Nice France; ^2^ Centre Oscar Lambret Lille France; ^3^ Centre d'Oncologie et de Radiothérapie de Haute Énergie du Pays Basque Bayonne France; ^4^ Institut Andrée Dutreix Dunkerque France; ^5^ Elsan Hôpital Privé Océane Vannes France; ^6^ Centre Hospitalier Bretagne Atlantique Vannes France; ^7^ Clinique Sainte Catherine Avignon France; ^8^ Department of Medical Affairs Merck Biopharma Lyon France; ^9^ Merck KGaA Darmstadt Germany; ^10^ Department of Drug Development and Innovation (D^3^i) Institut Curie Saint‐Cloud France; ^11^ INSERM U900 Research Unit Institut Curie Saint‐Cloud France; ^12^ Espace Technologique, Bat. Discovery Paris‐Saclay University Paris France

**Keywords:** 5‐fluorouracil, carboplatin, cetuximab, cisplatin, head and neck neoplasms, palliative care

## Abstract

**Background:**

Cetuximab plus platinum‐based therapy (PBT) followed by cetuximab maintenance until progression (EXTREME) is a guideline‐recommended first‐line treatment option in recurrent/metastatic squamous cell carcinoma of the head and neck (R/M SCCHN). DIRECT (Dose Intensity RElative to CeTuximab) was the first phase 4 observational study evaluating EXTREME administration in the real‐world setting.

**Aims:**

The primary aim of this study was to assess the relative dose intensity of cetuximab in patients with R/M SCCHN treated with first‐line cetuximab according to the EXTREME regimen.

**Methods and results:**

Patients were ≥18 years old and eligible to receive cetuximab/PBT. Primary endpoint was cetuximab relative dose intensity (RDI). Of prospectively enrolled patients (*n* = 157), 119 received ≥1 cycle of EXTREME. Practices differing from the EXTREME trial were 5‐fluorouracil omission (14%), maintenance cetuximab given every other week (54%), prior cetuximab, disease‐free interval <6 months. 64% of patients reached cetuximab RDI ≥80%; mean cetuximab RDI was 88%. 46% of patients received maintenance cetuximab (mean RDI, 91%). Median progression‐free survival and overall survival were 4.5 and 9.4 months. No new/unexpected safety findings were observed.

**Conclusions:**

The DIRECT study showed that first‐line cetuximab plus PBT was a feasible, beneficial first‐line treatment regimen in patients with R/M SCCHN in the real‐world setting.

## 1 INTRODUCTION

Recurrent and/or metastatic squamous cell carcinoma of the head and neck (R/M SCCHN) has a poor prognosis.^1^ Cetuximab plus platinum‐based chemotherapy followed by cetuximab maintenance (EXTREME) was the first regimen to yield significant survival benefits over chemotherapy alone in the first‐line treatment of R/M SCCHN.^2^, ^3^, ^4^, ^5^ This regimen, which is currently an established first‐line treatment option for patients with R/M SCCHN, is composed of ≤6 cycles of a platinum‐based chemotherapy (cisplatin/carboplatin +5‐fluorouracil [5‐FU]) with the anti‐epidermal growth factor receptor (EGFR) monoclonal antibody cetuximab, followed by maintenance cetuximab therapy until progressive disease (PD).^2^ The EXTREME regimen resulted in a median overall survival (OS) improvement of nearly 3 months over chemotherapy alone (10.1 vs. 7.4 months), and an overall response rate of 36% versus 20% in the chemotherapy‐alone arm.^2^ Maintenance therapy is a standard component of the EXTREME regimen, and international guidelines recommend continuing cetuximab treatment until disease progression for patients with at least stable disease after combination treatment with chemotherapy.^2^, ^5^, ^6^ The treatment landscape for patients with R/M SCCHN is rapidly evolving as multiple first‐line treatment options become available, including immune checkpoint inhibitors as monotherapy as well as in combination with chemotherapy. Although pembrolizumab has demonstrated promising efficacy in patients with R/M SCCHN,^7^ additional data are required to understand the clinical benefit of the available treatment options for different patient populations.

The phase 4 DIRECT trial is the first observational, prospective study to characterize physicians' treatment practices and patient adherence with the EXTREME regimen in the first‐line R/M SCCHN setting.^8^ This study enrolled largely unselected patients, thereby reflecting the real‐world population observed in clinical practice. Patients could be treated with the EXTREME regimen as described in the pivotal study or, at the physicians' discretion, with an adapted version of the EXTREME regimen to address individual patients' conditions. Thus, the findings of this observational study shed light on how the EXTREME regimen is applied in the real‐world setting.

## 2 METHODS

### 2.1 Trial design and patients

DIRECT (EMR 62202‐556) was a phase 4, observational, longitudinal, multicenter, noncomparative study to assess the relative dose intensity (RDI) of cetuximab in patients with R/M SCCHN undergoing first‐line treatment with the EXTREME regimen across France. The trial spanned 21 months, which included a 6‐month recruitment period and a maximum follow‐up period of 12 months. Patients were accrued prospectively (before beginning cetuximab treatment). Any individual ≥18 years of age with histologically proven R/M SCCHN and eligibility to receive the EXTREME regimen could participate in this study. Those who required an adaptive EXTREME regimen (e.g., patients with cardiovascular disorders [a contraindication to 5‐FU]) could also participate at physicians' discretion. Exclusion criteria consisted of concomitant participation in an interventional trial, known allergic reaction to one of the treatment components, and factors that impinged on the patient's ability to maintain adherence throughout the study. Also excluded were patients treated with cetuximab according to the scheme of the EXTREME study who had received <1 complete cycle of chemotherapy in combination with cetuximab, patients with nasopharyngeal carcinoma, and patients with a contraindication in accordance with the respective label, except for that of 5‐FU. Finally, it is worth noting that patients were recruited before any anti‐programmed death‐(ligand) 1 (PD‐[L]1) therapies were available for the R/M SCCHN population.

In accordance with European regulations, French observational studies do not require review or approval from an institutional review board or institutional ethics committee. Nevertheless, these studies are not exempt from scientific opinion or ethical and legal authorization.

### 2.2 Treatment

Treatment was conducted per physician's choice and largely per label specifications for cetuximab and according to the EXTREME protocol. The EXTREME label consists of cetuximab (loading dose of 400 mg/m^2^ as a 2‐h intravenous [IV] infusion and then 250 mg/m^2^ as a 1‐h IV infusion per week) + cisplatin (100 mg/m^2^ on day 1) or carboplatin (area under the curve of 5 mg/ml/min as a 1‐h IV infusion on day 1) + 5‐FU (1000 mg/m^2^/day for 4 days) every 3 weeks for a maximum of six cycles, with the intention to continue cetuximab until PD. The only adaptations were to omit 5‐FU due to preexisting cardiovascular disorders, to administer cetuximab every 2 weeks in the maintenance phase (at a dose of 500 mg/m^2^), and to enroll patients with prior cetuximab treatment in the locally advanced (LA) setting. An exception to the physician's discretion was to exclude patients who had received a taxane as part of the first‐line regimen.

### 2.3 Outcome assessment

The primary objective of this study was to describe the use of the EXTREME regimen in a real‐world clinical setting. The selected method of measurement was the cetuximab RDI (defined as the ratio of actual cumulative dose received by patients to the planned dose), and the primary study endpoint was the percentage of patients with a cetuximab (or chemotherapy) RDI of ≥80%. Mean RDI is an indicator of whether patients can successfully complete the recommended regimen in the real world.^9^ A key secondary objective was the assessment of cetuximab (and, when applicable, chemotherapy) RDI by treatment phase (combination and maintenance). Additional secondary objectives included determination of the incidence of skin reactions related to cetuximab (and cetuximab RDI) based on the National Cancer Institute Common Terminology Criteria for Adverse Events v4.03, analysis of the impact that management of skin reactions may have on cetuximab RDI, and determining reasons why patients may discontinue or interrupt treatment and thus achieve a lower RDI. A complementary statistical analysis was also performed to determine survival outcomes at 12 months, including progression‐free survival (PFS) and OS. Finally, patients were followed up until treatment discontinuation or for a maximum of 12 months after their first visit. Per an amendment to the protocol, the status of all patients who discontinued the study within <1 year of the inclusion visit was retrospectively recorded as 1 year.

### 2.4 Statistics

On the basis of the EXTREME study results (84% of patients had a cetuximab RDI of ≥80%),^2^ it was estimated that, of 150 enrolled patients, 135 would be evaluable for a cetuximab RDI of ≥80%. The full analysis sample was defined as all patients who received ≥1 dose of cetuximab in addition to the loading dose. The statistical safety analysis consisted of all patients who received ≥1 dose of cetuximab (i.e., the loading dose). Patient status was evaluated at 12 months; PFS and OS analyses were performed using Kaplan–Meier curves. Exploratory subgroup analyses were performed using univariate statistical tests (χ^2^, Fisher exact, *t* or Wilcoxon, log‐rank). If indicated, multivariate analysis was performed using Cox regression. Statistical analysis was performed using SAS software (SAS Institute, Cary, NC).

## 3 RESULTS

### 3.1 Patient population

From November 2012 to June 2015, the DIRECT study prospectively enrolled and observed 169 patients with previously untreated R/M SCCHN. A total of 157 patients received at least the initial loading dose of cetuximab; this group is the key analyzed population in this report. Of these patients, 96 had received chemotherapy in the LA setting before entering DIRECT, and 30 patients had previously received cetuximab. Detailed information regarding prior treatments is presented in Table 1.

**TABLE 1:** Baseline characteristics and previous therapy of the prospective patients enrolled in DIRECT

Parameter	Prospective patients who received at least the loading dose of cetuximab (*N* = 157)	
Age, years	*N* available (%)	157 (100)
	Mean (SD)	59.8 (7.7)
	Range	41–78
Sex	*N* available (%)	157 (100)
	Male	134 (85.4)
	Female	23 (14.6)
BMI, kg/m^2^	*N* available (%)	153 (97.5)
	Mean (SD)	21.7 (4.6)
ECOG PS	*N* available (%)	141 (89.8)
	0	35 (24.8)
	1	80 (56.7)
	2	25 (17.7)
	4	1 (0.7)
Disease characteristics	*N* available (%)	156 (99.4)
	Locoregionally recurrent	76 (48.7)
	Recurrent with metastases	63 (40.4)
	Metastatic at first presentation	17 (10.8)
Disease‐free interval before study entry	Locoregionally recurrent; N available (%)	70^a^ (44.6)
	Locoregionally recurrent (<6 months)	25 (35.7)
	Locoregionally recurrent (≥6 months)	45 (64.3)
	Recurrent with metastases; *N* available (%)	63 (40.1)
	Recurrent with metastases (<6 months)	36 (57.1)
	Recurrent with metastases (≥6 months)	27 (42.9)
Primary tumor site	*N* available (%)	156 (99.4)
	Oral cavity	46 (29.5)
	Oropharynx	45 (28.8)
	Hypopharynx	35 (22.4)
	Larynx	29 (18.6)
	Other	1 (0.6)
Previous treatments	*N* available (%)	140 (89.2)
	Surgery + RT + chemotherapy	40 (28.6)
	RT + chemotherapy	29 (20.7)
	Surgery + RT	29 (20.7)
	RT + chemotherapy + cetuximab	13 (9.3)
	Surgery + RT + chemotherapy + cetuximab	11 (7.9)
	RT	6 (4.3)
	RT + cetuximab	4 (2.9)
	Surgery	4 (2.9)
	Chemotherapy	1 (0.7)
	Chemotherapy + cetuximab	1 (0.7)
	Surgery + chemotherapy	1 (0.7)
	Surgery + RT + cetuximab	1 (0.7)
TNM staging system	*N* available (%)	148 (94.3)
	Stage I	4 (2.7)
	Stage II	12 (8.1)
	Stage III	27 (18.2)
	Stage IVa	36 (24.3)
	Stage IVb	51 (34.5)
	Stage IVc	18 (12.2)

Abbreviations: BMI, body mass index; ECOG PS, Eastern Cooperative Oncology Group performance status; RT, radiotherapy.

^a^ Timing of relapse for locoregionally recurrent patients (<6 vs. ≥6 months) is missing for six patients.

Of the 157 patients, 139 patients entered the study with recurrent disease (locoregional only, *n* = 76; recurrent with metastasis, *n* = 63), 17 patients had metastasis upon initial diagnosis, and one patient's status upon enrollment was missing (Table 1). A total of 18.4% of patients had an Eastern Cooperative Oncology Group performance status (ECOG PS) of ≥2; inclusion of these patients was based on physicians' discretion. Furthermore, 29.9% of patients in DIRECT were ≥65 years of age. Additional baseline characteristics of the DIRECT population are presented in Table 1.

### 3.2 Treatment exposure

In DIRECT, among the 157 patients who received the cetuximab loading dose, 140 also received ≥1 additional dose of cetuximab, and 17 patients discontinued treatment following the loading dose. The chemotherapy regimens received and the number of patients who received each regimen are described in Table 2. A total of 39.0% of patients began with a carboplatin‐based regimen; 61.0% received cisplatin‐based treatment. Nineteen patients (13.5%) switched from cisplatin‐ to carboplatin‐based therapy, and two patients (1.4%) switched from carboplatin‐ to cisplatin‐based treatment (Table 2). The median cumulative dose was 245 mg/m^2^ for cisplatin (± 5‐FU), 1067 mg/m^2^ for carboplatin (± 5‐FU), 12 005 mg/m^2^ for 5‐FU, 179 and 658 mg/m^2^ for cisplatin and carboplatin (± 5‐FU) before the switch. The mean RDI was 79.1 ± 25.1% (cisplatin ±5‐FU), 81.9 ± 27.5% (carboplatin ±5‐FU), 82.7 ± 17.8% (5‐FU), and 80.4 ± 27.6% and 86.9 ± 23.3% (patients having switched from cisplatin to carboplatin or carboplatin to cisplatin, respectively).

**TABLE 2:** Chemotherapy regimens administered in combination with cetuximab during the combination phase of the DIRECT trial

Patients who received any chemotherapy regimen	
*n* (%)	157 (100.0)
Cisplatin	7 (4.5)
Cisplatin + 5‐FU	87 (55.4)
Carboplatin	15 (9.6)
Carboplatin + 5‐FU	47 (29.9)
Cisplatin + carboplatin + 5‐FU	1 (0.6)
Patients with known chemotherapy treatment sequence	
Total	141 (89.8)
Patients who did not undergo a regimen switch, n (%)	
Cisplatin	3 (2.1)
Cisplatin + 5‐FU	64 (45.4)
Carboplatin	16 (11.3)
Carboplatin + 5‐FU	37 (26.2)
Cisplatin + carboplatin + 5‐FU	1 (0.6%)
Patients who underwent a regimen switch, n (%)	
Cisplatin + 5‐FU → carboplatin	3 (2.1)
Cisplatin + 5‐FU → carboplatin + 5‐FU	13 (9.2)
Cisplatin → carboplatin	3 (2.1)
Carboplatin + 5‐FU → cisplatin	1 (0.7)
Carboplatin + 5‐FU → cisplatin + 5‐FU	1 (0.7)

Abbreviation: 5‐FU, 5‐fluorouracil.

Note: Arrow symbolizes a chemotherapy switch.

Cetuximab dose delays occurred in 39.3% of patients, and dose reductions occurred in 14.3%. These changes were primarily reported during the combination phase (dose delays, 34.3% and 25.0%; dose reductions, 9.3% and 11.1%, in the combination vs the maintenance phase, respectively).

During the combination phase, the mean cetuximab RDI remained high (87.6% ± 16.9%), and 64.3% of patients had a cetuximab RDI of ≥80%. The mean RDI with chemotherapy was 79.1% for cisplatin, 81.9% for carboplatin, and 82.7% for 5‐FU (Table 3).

**TABLE 3:** Mean RDI during treatment with cetuximab plus platinum‐based chemotherapy, followed by maintenance cetuximab until progression or unacceptable toxicity

Therapy	Mean RDI ± SD (%): combination phase	Mean RDI ± SD (%): maintenance phase
Cisplatin	79.1 ± 25.1	NA
Carboplatin	81.9 ± 27.5	NA
5‐FU	82.7 ± 17.8	NA
Cetuximab	87.6 ± 16.9	91.4 ± 15.7

Abbreviations: 5‐FU, 5‐fluorouracil; NA, not applicable; RDI, relative dose intensity.

Seventy‐two patients (45.9%) received ≥1 dose of cetuximab in the maintenance setting, and the median duration of maintenance therapy was 14.2 weeks. In this cohort, the mean cetuximab RDI was 91.4% ± 15.7% (Table 3) and ≥80% in 57 (85.1%) of the 67 patients for whom RDI data were available. A total of 45.8% of patients (n = 33) who received maintenance therapy were treated with weekly cetuximab, and 54.2% (*n* = 39) received cetuximab every 2 weeks.

Of the 153 patients who discontinued from the study, the following reasons for discontinuation were available: 79 (51.6%) due to PD, 39 (25.5%) due to death, 6 (3.9%) due to skin adverse events (AEs), 3 (2.0%) due to other treatment‐related AEs, 20 (13.1%) listed as other reasons, and 6 (3.9%) listed as missing or lost to follow up. Carboplatin‐receiving patients most commonly discontinued chemotherapy due to PD (33.3%), toxicity (20.8%), and completion of the planned regimen (16.7%). Cisplatin‐receiving patients most commonly discontinued chemotherapy due to toxicity (39.6%), PD (27.1%), and completion of the planned regimen (20.8%). The median number of chemotherapy cycles was 4.

### 3.3 Safety of treatment with cetuximab

Among the 157 patients, the total incidence of skin reactions of any grade during treatment was 70.7%, and the most common dermatologic AE was papulopustular eruption/acne‐like rash, which occurred in 91 patients (58.0%), followed by xerosis or dry skin in 49 patients (31.2%) (Table 4). Additionally, the incidence rate of infusion‐related reactions was 1.9%.

**TABLE 4:** Rate of skin reactions in the prospective population of DIRECT

*N* = 157	Patients, %	
	Grade 1/2	Grade 3/4
Total	68.8	7.6^a^
Papulopustular eruption or acne‐like rash	55.4	3.8
Xerosis or dry skin	25.5	2.5
Skin fissures	21.7	1.9
Paronychia or periungual lesions	5.1	1.3
Other	5.1	0.6
Not documented	3.8	0.0

^a^ All grade 3 except 1 case (0.6%) of grade 4 papulopustular eruption or acne‐like rash.

Only 12 patients (7.6%) experienced a grade ≥3 skin AE, and six and four patients (3.8% and 2.5%, respectively) experienced grade ≥3 papulopustular eruption/acne‐like rash and xerosis/dry skin, respectively. Regardless of the treatment phase, the incidence of skin reactions did not significantly vary according to the RDI rate.

Preventive treatments were administered to 22.9% of patients to avoid the occurrence of skin reactions. Overall, the most frequently reported prescribed preventive skin management treatments were oral antibiotics (cyclins; 16.6%) and fatty emollients (15.9%). All others (e.g., level III dermocorticosteroids, antiseptics) were each given to <5% of patients. Half of patients (48.4%) received reactive skin treatments, and the most common were fatty emollients (29.9%) and oral antibiotics, such as cyclins (26.8%); furthermore, level III dermocorticosteroids were used in 11.5% of patients. A minority of patients (*n* = 8 [5.1%]) experienced grade ≥3 skin AEs, for which they received reactive skin treatment. RDI (*n* = 48 with RDI of <80% and *n* = 82 with RDI of ≥80%) was not significantly associated with the proportion of patients receiving ≥1 preventive or reactive skin treatment (preventive: 28.1% and 71.9% in patients with RDU <80% and ≥80%, respectively; p = 0.235 by χ^2^ test; reactive: 31.9% and 68.1% in patients with RDU <80% and ≥80%, respectively; p = 0.205 by χ^2^ test).

A total of 56 patients (35.7%) experienced other treatment‐related AEs, either nonhematologic (26.8%) or hematologic (21.7%). The most frequently reported nonhematologic AEs were “other” AEs (14.0%, mainly mucositis [90.9% of the 22 patients who experienced an AE categorized as “other”]), followed by asthenia (10.2%), hypomagnesemia (6.4%), hypokalemia (7.0%), and vomiting (6.4%). The most common hematologic AEs were anemia (10.8%), neutropenia (8.9%), and thrombopenia (5.7%). The most common grade ≥3 “other” AEs occurred in 17.2% of patients and mainly included neutropenia (5.7%), other AEs (mucositis and digestive disorders [2.5%]), and hypokalemia (3.2%).

### 3.4 Efficacy

**FIGURE 1:**
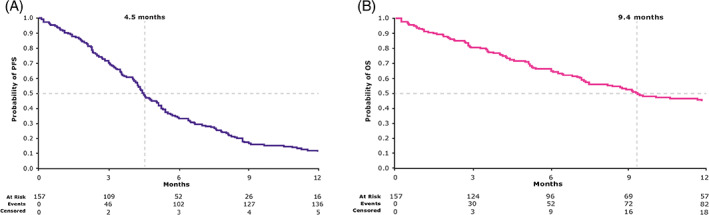
**(**A) Progression‐free survival of the prospective population of DIRECT (patients who received ≥1 dose of cetuximab; *N* = 157). (B) Overall survival of the prospective population of DIRECT (patients who received ≥1 dose of cetuximab; *N* = 157)

Median follow‐up was 8 months (range, 0–29 months). The 12‐month PFS rate was 11.3% (95% CI, 6.9%–17.0%), and the 12‐month OS rate was 44.2% (95% CI, 35.9%–52.1%). Median PFS and OS were 4.5 months (95% CI, 4.1–5.1 months) and 9.4 months (95% CI, 7.2–13.3 months), respectively (Figure 1(A), (B)).

Several subgroups of patients were further analyzed in a univariate Cox model for potential prognostic characteristics. Among 157 patients (110 were <65 and 47 were ≥65 years of age), the 12‐month OS rate (41.0% and 51.4%, respectively; p = 0.3420 by log‐rank test) was not found to be significantly affected by age distribution categories. Among 133 patients with recurrent disease for whom data were available, 61 patients had a known disease‐free interval (DFI) of <6 months, and 72 patients were disease free for ≥6 months before entering DIRECT. DFI was defined as the time from last cisplatin in definitive phase (LA SCCHN) to the time of progression in 1 L R/M. The length of the DFI before enrollment did not appear to be prognostic for the 12‐month OS rate (44.3% and 42.1% for patients who experienced a DFI of <6 vs. ≥6 months, respectively; p = 0.8580 by log‐rank test) (Table 5; multivariable analysis shown in Table 6). Finally, previous cetuximab treatment in the LA setting was not found to significantly affect the 12‐month OS rate, although a trend was observed toward better survival for patients who received cetuximab in the LA setting (59.6% vs. 41.0% for patients previously treated with cetuximab vs. not; p = 0.0771 by log‐rank test) (Table 5).

**TABLE 5:** Univariate analysis of the potential prognostic value of previous cetuximab treatment, disease‐free survival, and maintenance cetuximab schedule in the prospective population

Subgroup		12‐month OS rate, *n* (%)	HR (95% CI)	p Value
Previous cetuximab treatment	No (*n* = 127)	55 (41.0)	0.550 (0.284‐1.067)	0.077
	Yes (*n* = 30)	20 (59.6)		
Disease‐free interval	<6 months (*n* = 61)	30 (44.3)	0.958 (0.599‐1.533)	0.858
	≥6 months (*n* = 72)	32 (42.1)		
Maintenance cetuximab schedule	Weekly (*n* = 33)	21 (62.6)	NA	0.197
	Every 2 weeks (*n* = 39)	31 (77.0)		

Abbreviations: NA, not available; OS, overall survival.

**TABLE 6:** Multivariable analysis for prognostic value of 12‐month OS rate in DIRECT

Variable	p Value	Hazard ratio	95% HR confidence limits	
Woman versus man	0.2056	0.543	0.211	1.397
≥65 versus <65 years	0.3884	0.743	0.378	0.388
ECOG PS 2–4 versus 0–1	0.0014	2.830	1.496	0.001
Initial diagnosis ≥12 versus <12 months	0.9052	0.959	0.482	0.905
Oropharynx versus oral cavity	0.7855	1.104	0.543	0.786
Hypopharynx versus oral cavity	0.9960	1.002	0.443	0.996
Larynx versus oral cavity	0.6947	1.179	0.518	0.695
T3/4 versus T1/2^a^	0.2152	0.688	0.380	0.215
N2/3 versus N0/1^a^	0.5500	0.841	0.477	0.550
M1 versus M0	0.6598	1.254	0.458	0.660
Metastatic progression versus relapse	0.8298	1.065	0.599	0.830
Free interval ≥6 versus <6 months	0.3950	0.744	0.376	0.395

Abbreviations: ECOG PS, Eastern Cooperative Oncology Group performance status; HR, hazard ratio; OS, overall survival.

^a^ Stage at initial diagnosis.

Additionally, 12‐month PFS and OS rates were not significantly different among patients who received cetuximab as maintenance therapy every week (*n* = 33) versus every 2 weeks (*n* = 39), although a trend was observed toward better survival with the every‐2‐weeks schedule (12‐month PFS rate: 18.2% vs. 27.5%; p = 0.22 by log‐rank test; 12‐month OS rate: 62.6% vs. 77.0%; p = 0.20 by log‐rank test) (Table 5). A univariate analysis found no prognostic value for the presence of metastatic progression (*n* = 80) versus locoregional recurrence only (*n* = 76) for survival (12‐month OS rate: 46.4% vs. 41.0%; p = 0.48 by log‐rank test). OS was not statistically significantly different between patients with RDI <80% and patients with RDI ≥80%; p = 0.76 by log‐rank test.

## 4 DISCUSSION

The DIRECT study was a phase 4, observational, longitudinal, confirmatory study of the real‐world practices and outcomes when using the EXTREME regimen or a modified version of the EXTREME regimen (at the physicians' discretion) in the first‐line treatment of patients with R/M SCCHN. This study was initiated before therapies with immune checkpoint inhibitors were available for patients with R/M SCCHN. Based on recent data demonstrating the efficacy of pembrolizumab in R/M SCCHN among patients with a combined positive score (CPS) ≥1 for PD‐L1 expression,^7^ pembrolizumab alone or in combination with platinum‐5‐FU has become a first‐line treatment option for this patient subpopulation. EXTREME and a cetuximab and platinum‐based regimen remain the standard for patients with CPS <1. Additional data are needed to determine the optimal sequence of treatment especially for R/M patients with disease that requires a rapid response.

The primary objective of the DIRECT study was to measure RDI in the real‐world setting as an indicator of the feasibility and tolerability of treatment with cetuximab‐ and platinum‐based chemotherapy followed by cetuximab maintenance therapy until PD (EXTREME regimen). PFS and OS were also assessed. Although the DIRECT study is not directly comparable to the EXTREME study, the proportion of patients who received maintenance therapy and the recorded median PFS and OS were comparable in both trials.

Baseline characteristics of the prospective DIRECT patient population who received at least the loading dose of cetuximab (*n* = 157) indicated that almost 20% of the real‐world patient population that received cetuximab plus platinum‐based therapy (PBT) had an ECOG PS of ≥2. Furthermore, nearly 30% of patients in DIRECT were ≥65 years of age. Finally, almost half of the patients in the DIRECT population had locoregional recurrence only (i.e., without metastatic disease). Hence, overall, the population in the DIRECT study who received at least the loading dose of cetuximab may have had a slightly worse prognosis compared with the population enrolled in the cetuximab‐containing arm of the EXTREME clinical trial. The patient population of the DIRECT study is likely more representative of the overall European population with R/M SCCHN in the real‐world setting. This observation highlights the importance of this study, as most patients with R/M SCCHN in Europe are treated with the EXTREME regimen in the first line.^10^, ^11^

Cetuximab RDI measurements suggest good adherence in combination with platinum‐based chemotherapy in the real world, although the proportion of patients with an RDI ≥80% was lower in the present study than in EXTREME (64% vs. 84%).^2^ Additionally, most patients received the full or close to the full planned dose of cetuximab in the maintenance phase, which is consistent with the findings of EXTREME (RDI ≥80% in 85% vs. 82% of patients in DIRECT vs. EXTREME).^2^ Maintenance therapy is a standard component of the EXTREME regimen, and it is thought to prolong response and take advantage of patients' responsiveness to anti‐EGFR therapy beyond the six cycles of combination treatment. In the DIRECT study, nearly half of the patients did not have PD at the end of the combination phase and thus entered the maintenance phase. Although comparison of the two studies warrants caution, median PFS and OS were similar in DIRECT versus EXTREME (PFS, 4.5 vs. 5.6 months; OS, 9.4 vs. 10.1 months),^2^ suggesting that the efficacy of a first‐line cetuximab‐ and platinum‐based chemotherapy regimen may not be substantially different in a broader patient population with R/M SCCHN and when a lower‐than‐planned number of chemotherapy cycles is given. Specifically, patients in DIRECT completed a median of four cycles of platinum‐based chemotherapy and thus started maintenance therapy earlier than patients in the cetuximab arm of EXTREME (median of five cycles^2^), which had little impact on OS or cetuximab RDI.

Several interesting observations in the real‐world treatment practices used in the DIRECT study were made. For example, patients were unselected beyond age and ECOG PS; nearly 20% of patients had an ECOG PS of ≥2. Furthermore, a subgroup analysis of survival suggested a benefit with cetuximab plus PBT, irrespective of whether patients had a DFI of <6 or ≥6 months before study entry. Additionally, previous cetuximab therapy in the LA setting did not appear to significantly affect survival. A total of 14.0% of patients in the DIRECT study were treated without 5‐FU, and more than half of the patients received cetuximab every 2 weeks during the maintenance phase. The similar survival results compared with the EXTREME trial suggest that a broad patient population with R/M SCCHN may benefit from first‐line cetuximab plus platinum‐based chemotherapy followed by cetuximab maintenance. Additionally, although the EXTREME regimen should be used according to the recommended dosing, adaptations to the chemotherapy regimen, dosing, number of cycles, and scheduling of this regimen that are not detrimental to survival benefit may be permissible. Additional prospective studies are needed to confirm these findings.

The recently published GLANCE H&N study examined global treatment patterns and real‐world outcomes among patients with R/M SCCHN. However, it should be noted that the eligibility period for this trial (2011–2013) occurred at a time when cetuximab was not reimbursed for the treatment of SCCHN in the United Kingdom and some of the other included countries, and only about 28% of patients in the GLANCE H&N study received a cetuximab‐based combination regimen. Because of subsequent changes in the reimbursement criteria for cetuximab since the GLANCE H&N study, the DIRECT study may reflect a more realistic view of real‐world outcomes that are in alignment with current reimbursement guidelines.^12^ The safety analysis of the DIRECT study also indicated no new or surprising rates of grade ≥3 skin reactions and grade 3 or 4 AE rates. Overall, the results of the DIRECT study demonstrated that cetuximab plus platinum‐based chemotherapy was feasible and tolerable.

## 5 CONCLUSION

The DIRECT study provided real‐world support for use of the EXTREME regimen in everyday clinical practice. The DIRECT study's outcomes (PFS, OS, safety profile) were similar to those observed in the cetuximab‐containing arm of EXTREME, although a distinct and more inclusive patient population was enrolled, including patients with DFI <6 months since the last platinum treatment, patients with prior cetuximab treatment, and patients with contraindications to 5‐FU. Nearly 50% of patients in DIRECT were able to complete the combination phase and thus continued to receive maintenance therapy. Additionally, the DIRECT study identified a low rate of treatment interruptions and dose reductions, the majority of which occurred in the combination therapy phase. In conclusion, the DIRECT study demonstrated that first‐line cetuximab plus platinum‐based chemotherapy, including cetuximab maintenance therapy, was a feasible and beneficial treatment regimen in patients with R/M SCCHN in the everyday clinical setting.

## CONFLICT OF INTEREST

Joël Guigay has served on advisory boards for AstraZeneca, Bristol Myers Squibb, Innate Pharma, and Merck KGaA and has received grants for research from GSK, Bristol Myers Squibb, Chugai, and Merck KGaA. Audrey Seronde is an employee of Merck Santé SAS, Lyon, France, an affiliate of Merck KGaA, Darmstadt, Germany. Jeltje Schulten is an employee of Merck KGaA, Darmstadt, Germany. Christophe Le Tourneau has an advisory role for Merck KGaA.

## AUTHOR CONTRIBUTIONS

All authors had full access to the data in the study and take responsibility for the integrity of the data and the accuracy of the data analysis. *Conceptualization*, J.G., C.L.T., A.S., J.S.; *Methodology*, J.G., C.L.T., A.S., J.S.; *Validation*, E.C.; *Investigation*, J.G., E.C., G.L., M.R., J.‐P.W., E.B., M.A., F.P., C.L.T.; *Formal Analysis*, E.C.; *Resources*, A.S., J.S.; *Writing ‐ Original Draft*, J.G., E.C., G.L., M.R., J.‐P.W., E.B., M.A., A.S., J.S., F.P., C.L.T.; *Writing ‐ Review and Editing*, J.G., E.C., G.L., M.R., J.‐P.W., E.B., M.A., A.S., J.S., F.P., C.L.T.; *Visualization*, J.G., E.C., G.L., M.R., J.‐P.W., E.B., M.A., A.S., J.S., F.P., C.L.T.; *Supervision*, J.G., C.L.T.; *Project Administration*, A.S., J.S.; *Funding Acquisition*, A.S., J.S.

## ETHICAL STATEMENT

In accordance with European regulations, French observational studies do not require review or approval from an institutional review board or institutional ethics committee. Nevertheless, these studies are not exempt from scientific opinion or ethical and legal authorization. Written informed consent was obtained from all study participants.

## ROLE OF FUNDING SOURCE

Merck Santé SAS, Lyon, France, an affiliate of Merck KGaA, Darmstadt, Germany, fully funded the study and designed the protocol and amendments in discussion and agreement with the coordinating investigators. Data were interpreted by Merck KGaA and the coordinating investigators. The final decision to submit for publication was made by the coordinating investigators.

## Data Availability

Any requests for data by qualified scientific and medical researchers for legitimate research purposes will be subject to Merck's Data Sharing Policy. All requests should be submitted in writing to Merck's data sharing portal (https://www.merckgroup.com/en/research/our-approach-to-research-and-development/healthcare/clinical-trials/commitment-responsible-data-sharing.html). When Merck has a co‐research, co‐development, or co‐marketing or co‐promotion agreement, or when the product has been out‐licensed, the responsibility for disclosure might be dependent on the agreement between parties. Under these circumstances, Merck will endeavor to gain agreement to share data in response to requests.
